
JOURNAL INFORMATION
==============================
NLM Title Abbreviation: Wirtschaftsdienst
Journal ID: Wirtschaftsdienst
NLM Journal ID: 101086612

Wirtschaftsdienst (Hamburg, Germany : 1949)

ISSN: 0043-6275

ARTICLE INFORMATION
==============================
PMCID: PMC7174818
PMID: 32336808
DOI: 10.1007/s10273-020-2637-z
Article ID: 2637
Article version: 1
Subjects: Ökonomische Trends

Szenarien einer globalen Rezession

Global recession scenarios

Vöpel Henning voepel@hwwi.org

grid.435054.3 0000 0001 2227 8589 HWWI, Oberhafenstraße 1, 20097 Hamburg, Deutschland
Electronic publication date: 2020 Apr 22
Print publication date: 2020
Volume: 100
Issue: 4
First page: 303
Last page: 304
Copyright: © Der/die Autor(en) 2020
License: Open Access Dieser Artikel wird unter der Creative Commons Namensnennung 4.0 International Lizenz veröffentlicht, welche die Nutzung, Vervielfältigung, Bearbeitung, Verbreitung und Wiedergabe in jeglichem Medium und Format erlaubt, sofern Sie den/die ursprünglichen Autor(en) und die Quelle ordnungsgemäß nennen, einen Link zur Creative Commons Lizenz beifügen und angeben, ob Änderungen vorgenommen wurden.
Die in diesem Artikel enthaltenen Bilder und sonstiges Drittmaterial unterliegen ebenfalls der genannten Creative Commons Lizenz, sofern sich aus der Abbildungslegende nichts anderes ergibt. Sofern das betreffende Material nicht unter der genannten Creative Commons Lizenz steht und die betreffende Handlung nicht nach gesetzlichen Vorschriften erlaubt ist, ist für die oben aufgeführten Weiterverwendungen des Materials die Einwilligung des jeweiligen Rechteinhabers einzuholen.
Weitere Details zur Lizenz entnehmen Sie bitte der Lizenzinformation auf http://creativecommons.org/licenses/by/4.0/deed.de.
License URL: https://creativecommons.org/licenses/by/4.0/

issue-copyright-statement: © The Author(s) 2020

==============================

Innerhalb von nur wenigen Wochen hat sich das Coronavirus COVID-19 von einem anfänglichen regionalen Problem Chinas zur größten globalen Bedrohung der Weltkonjunktur und wohl einer der größten Rezessionen der letzten 100 Jahre entwickelt. Zugleich unterscheidet sich die Corona-Rezession wesentlich von „normalen" Rezessionen. Zum Schutz der öffentlichen Gesundheit haben fast alle großen Volkswirtschaften für weite Teile ihrer Wirtschaft einen Shutdown verhängt, um eine unkontrollierte Ausbreitung der Pandemie zu vermeiden, die angesichts knapper Kapazitäten in der intensivmedizinischen Versorgung viele Todesopfer fordern würde. Das plötzliche, behördlich verfügte Herunterfahren der Produktion stellt einen Angebotsschock dar, der sich durch Unterbrechungen der Produktions- und Lieferketten sowie einen nachgelagerten Nachfrageschock auf nahezu die gesamte Ökonomie ausweitet. Durch den Einbruch bei den Erlösen bei weiterlaufenden Fixkosten entstehen akute Liquiditätsprobleme mit der Gefahr von Insolvenzen, die größer wird, je länger der Shutdown andauert. Ein Exit aus dem Shutdown lässt sich nur in Abhängigkeit von den zu erwartenden Neuinfektionen vollziehen.

Verschiedene Dynamiken interagieren somit komplex miteinander, sodass der Exit keine zeitpunktbezogene Entscheidung, sondern ein optimaler Pfad der kontrollierten Öffnung ist. Da wenig über die Immunität der Bevölkerung und die Infektionswege des Coronavirus bekannt ist, lässt sich die Dauer des Shutdowns und der Grad seiner Lockerung zudem kaum prognostizieren, woraus hohe Unsicherheit über die konjunkturellen Folgen resultiert. Um die Wirtschaft für die Dauer des Shutdowns gewissermaßen „einzufrieren" oder in eine Art „künstliches Koma" zu versetzen, damit es nicht zu einer Insolvenzwelle und anderen Folgeschäden kommt, wurden umfangreiche staatliche Liquiditätshilfen zur Verfügung gestellt, die im Wesentlichen in direkten Transfers, Kreditlinien und Bürgschaften sowie Kurzarbeitergeld bestehen. Die restriktiven Maßnahmen wurden zum Teil als überzogen und wirtschaftlich schädlich kritisiert, jedoch zeigen die Beispiele anderer Länder wie China oder Italien, aber auch die Erfahrungen früherer Pandemien, beispielsweise der Spanischen Grippe von 1918/1919, dass eine unkontrollierte Pandemie am Ende teurer ist als die Maßnahmen zu ihrer Eindämmung (Correia et al., 2020; Barro et al., 2020).

Die Vollbremsung der Wirtschaft hat gravierende konjunkturelle und potenziell längerfristige Folgen, die angesichts der Unsicherheit über die epidemiologischen und ökonomischen Dynamiken nur in Szenarien dargestellt werden können. Die Bandbreite der aktuellen Konjunkturprognosen zeigt das Ausmaß der Prognoseunsicherheit (vgl. Tabelle 1). Das ifo Institut hat für unterschiedliche Szenarien ein Wachstum für Deutschland zwischen -7 % und -20 % berechnet. Bedeutsamer als die Punktprognose ist das dahinterliegende Konjunkturbild. Stilisiert kann man von einem „V", also einem schnellen starken Einbruch und einer ebenso schnellen Erholung, einem „U", also einer länger anhaltenden Talsohle, sowie einem „L", also einem geringeren Wachstumspfad nach der Krise, sprechen (vgl. Abbildung 1). Welcher dieser Verläufe eintreten wird, hängt wiederum von der Dauer des Shutdowns sowie den eingesetzten wirtschaftspolitischen Instrumenten ab. Das steile V lässt sich angesichts der derzeitigen Diskussion nahezu ausschließen. Ein ausgeprägtes U ist für den Fall zu erwarten, dass der Shutdown recht bald gelockert und die Wirtschaft stückweise wieder hochgefahren werden kann. In diesem Fall wären die getroffenen Maßnahmen geeignet, strukturelle Verwerfungen und Schäden zu vermeiden. Bei einem längeren und wiederholten Shutdown dürften Insolvenzen nicht zu vermeiden sein. Oder aber Unternehmen sehen sich durch die Überbrückungskredite deutlich höheren Verbindlichkeiten gegenüber, was zu einem geringeren Expansionspfad nach der Krise führen kann. Beides kann zu einem gegenüber dem Vorkrisenzustand niedrigeren Wachstumspfad führen. Neben den makroökonomischen Folgen dürften auch auf mikroökonomischer Ebene relevante Prozesse stattfinden, wie etwa ein beschleunigter Strukturwandel durch vorgezogene Marktkonsolidierung, aber auch das Ausscheiden kleinerer, weniger liquiditätsstarker und kreditwürdiger Anbieter. Solche Phänomene sowie Hysterese-Effekte

Tabelle 1
**Ausgewählte Konjunkturprognosen**

| | 2020 | 2021 |
|---|---|---|
| Sachverständigenrat | -2,8 | 3,7 |
| Gemeinschaftsdiagnose | -4,2 | 5,8 |
| Consensus Forecast | -4,3 | 3,8 |
| HWWI | -5,0 | 4,5 |

Abbildung 1
**Stilisierte Rezessionsverläufe V, U und L**

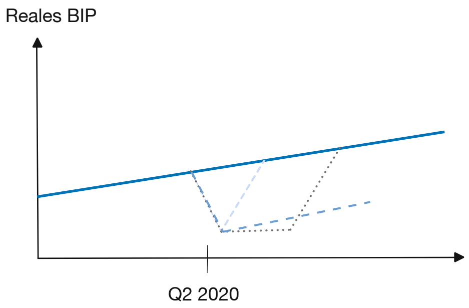

Quelle: eigene Darstellung.

werden maßgeblich durch die Dauer des Shutdowns und die Wahl der wirtschaftspolitischen Instrumente beeinflusst.

Derzeit ist ein ausgeprägteres U wahrscheinlich, da sich die Corona-Krise phasenverschoben verbreitet, angefangen in China, fortgesetzt in Europa und nun die USA erreichend. Produktions- und Lieferketten sowie Absatzmärkte bleiben dadurch länger gestört. Insbesondere in den USA könnte die Corona-Krise zu längerfristigen Verwerfungen führen. Da weniger Schutz durch Kranken- und Arbeitslosenversicherung und vor Kündigung besteht, können sich die Corona-Pandemie und die Rezession wechselseitig verschärfen und automatische Stabilisatoren weniger stützen. In den USA ist binnen einer Woche – noch am Anfang der Pandemie – die Arbeitslosigkeit um 6,6 Mio. gestiegen. Auch die Rückkehr der Pandemie über eine zweite Ansteckungswelle nach Lockerung des Shutdowns ist wahrscheinlich, zumindest nach den Erfahrungen von Ländern wie China oder Japan. Auch dies wird im Jahresverlauf 2020 die globale Konjunktur dämpfen. Schnell ansteigende Arbeitslosigkeit und Schuldenstände dürften gravierende Folgewirkungen auf die konjunkturelle Erholung haben.

Mittel- und langfristige Folgen der Corona-Krise sind ebenfalls wahrscheinlich. Mittelfristig drohen Folgekrisen, die vor allem mögliche Banken- und Staatsschuldenkrisen betreffen, inklusive möglicher Inflations- und Deflationsszenarien. In der Europäischen Union und insbesondere in der Eurozone dürften die asymmetrischen fiskalpolitischen Spielräume der Länder, auf die Krise zu reagieren, die Ungleichgewichte und die Divergenz beispielsweise in den Staatsschuldenquoten verstärken. Hinzu können ausfallende Kredite bei Banken kommen, die eine Banken- und Schuldenkrise auslösen können. Die Europäische Zentralbank hat mit dem Pandemie-Notfallkaufprogramm (Pandemic Emergency Purchase Programm [PEPP]) ein weiteres Instrument zur Liquiditätsversorgung und Stützung bereitgestellt. Eine indirekte Monetisierung auch von Staatsschulden ist diesem Instrument immanent. Darüber hinaus werden sogenannte Corona-Bonds als eine zweckgebundene und befristete Form von Eurobonds diskutiert, um eine gemeinsame und gesamtschuldnerische Finanzierungsmöglichkeit zu schaffen. Corona-Bonds als Alternative zu Finanzierungen über den Europäischen Stabilitätsmechanismus (ESM) und die Europäische Investitionsbank (EIB) sowie die Europäische Union für Kurzarbeitergeld (SURE) lassen sich auch über eine Versicherungslösung begründen. Die Corona-Krise trifft Europa exogen, ohne dass das Ausmaß für die einzelnen Länder vorher bekannt ist. In diesem Sinn handelt es sich um einen symmetrischen stochastischen Schock. Unter diesen Bedingungen würde man sich in einem Gedankenexperiment ex ante auf die gemeinsame Finanzierung über Corona-Bonds einigen. Über die Finanzierung der direkten Kosten der Corona-Krise hinaus wird es zwar kein „Wiederaufbauprogramm" geben müssen, denn der private und öffentliche Kapitalstock sind intakt, aber eine Art Konjunkturprogramm zur Wiederbelebung der europäischen und der globalen Wirtschaft. Denn negative Effekte multiplizieren sich über die Zeit, sodass mit Fortgang der Krise aus einer kurzen Rezession eine längere Depression resultieren kann.

Die Corona-Krise hat darüber hinaus potenziell Folgen für die Globalisierung. Langfristige Folgen können in Anpassungen der Unternehmen in ihren internationalen Produktions- und Lieferketten bestehen. Es ist zu erwarten, dass die Erfahrung der Verwundbarkeit von Just-in-time-Lieferketten zu einer stärkeren Diversifizierung, in Teilen auch Regionalisierung führen wird. Auch kann der Anteil der Staatswirtschaft steigen, zum einen durch etwaige Beteiligungen an Unternehmen infolge der Krisenmaßnahmen, zum anderen durch eine Re-Nationalisierung der Produktion von strategisch wichtigen Gütern, etwa bei Medizintechnik, Pharmazie oder Nahrungsmitteln, und diesbezüglich verstärkten Autarkiebestrebungen. In der Folge kann das die ohnehin vorhandenen Tendenzen einer De-Globalisierung noch verstärken mit entsprechenden Folgen für die internationalen Handelskosten. Die Corona-Krise kann schon heute als ein einschneidendes Ereignis gewertet werden, das die Weltwirtschaft über Jahre hinaus maßgeblich verändern wird.

Henning Vöpel
voepel@hwwi.org


Literatur

Barro R J Ursua J F Weng J The Coronavirus and the Great Infl uenza Pandemic: Lessons from the “Spanish Flu” for the Coronavirus’s Potential Effects on Mortality and Economic Activity NBER Working Paper 2020 26866
Consensus Forecast Umfrage April 2020 2020
Correia S Luck S Verner E Pandemics Depress the Economy Public Health Interventions Do Not: Evidence from the 1918 Flu 2020
Gemeinschaftsdiagnose Frühjahrsgutachten 2020 2020
HWWI aktualisierte Konjunkturprognose April 2020 2020
Sachverständigenrat zur Begutachtung der gesamtwirtschaftlichen Entwicklung (2020), Sondergutachten 2020.
